# MobiDB-lite 4.0: faster prediction of intrinsic protein disorder and structural compactness

**DOI:** 10.1093/bioinformatics/btaf297

**Published:** 2025-05-10

**Authors:** Mahta Mehdiabadi, Matthias Blum, Giulio Tesei, Sören von Bülow, Kresten Lindorff-Larsen, Silvio C E Tosatto, Damiano Piovesan

**Affiliations:** Department of Biomedical Sciences, University of Padova, 35131 Padova, Italy; European Molecular Biology Laboratory, European Bioinformatics Institute (EMBL-EBI), Wellcome Genome Campus, Hinxton, Cambridgeshire CB10 1SD, United Kingdom; Structural Biology and NMR Laboratory, Linderstrøm-Lang Centre for Protein Science, Department of Biology, University of Copenhagen, 2200 Copenhagen, Denmark; Structural Biology and NMR Laboratory, Linderstrøm-Lang Centre for Protein Science, Department of Biology, University of Copenhagen, 2200 Copenhagen, Denmark; Structural Biology and NMR Laboratory, Linderstrøm-Lang Centre for Protein Science, Department of Biology, University of Copenhagen, 2200 Copenhagen, Denmark; Department of Biomedical Sciences, University of Padova, 35131 Padova, Italy; Institute of Biomembranes, Bioenergetics and Molecular Biotechnologies, National Research Council (CNR-IBIOM), 70126 Bari, Italy; Department of Biomedical Sciences, University of Padova, 35131 Padova, Italy

## Abstract

**Motivation:**

In recent years, many disorder predictors have been developed to identify intrinsically disordered regions (IDRs) in proteins, achieving high accuracy. However, it may be difficult to interpret differences in predictions across methods. Consensus methods offer a simple solution, highlighting reliable predictions while filtering out uncertain positions. Here, we present a new version of MobiDB-lite, a consensus method designed to predict long IDRs and classify them based on compositional biases and conformational properties.

**Results:**

MobiDB-lite 4.0 pipeline was optimized to be ten times faster than the previous version. It now provides compactness annotations based on predicted apparent scaling exponent. The newly added features and disorder subclassifications allow the users to get a comprehensive insight into the protein’s function and characteristics. MobiDB-lite 4.0 is integrated into the MobiDB and DisProt databases. A version without the compactness predictor is integrated into InterProScan, propagating MobiDB-lite annotations to UniProtKB.

**Availability and implementation:**

The MobiDB-lite 4.0 source code and a Docker container are available from the GitHub repository: https://github.com/BioComputingUP/MobiDB-lite.

## 1 Introduction

Intrinsically disordered proteins and regions (IDPs/IDRs) are characterized by their lack of stable folded structures and their adoption of various rapidly interchanging conformations described by a conformational ensemble (Forman-Kay and Mittag 2013). Despite having structural flexibility, IDPs/IDRs exhibit local and global ordering, influencing their size, shape, interactions with other proteins, and overall biological function (Wright and Dyson 2015). These regions may be critical for the formation and dynamics of biomolecular condensates within cells (Pappu *et al.* 2023) and play a key role in physiological and pathological processes associated with misfolding and aggregation (Silva *et al.* 2017).

Many computational tools have been developed to predict disordered regions, but most were per-residue based, resulting in fragmented predictions that failed to accurately capture long IDRs (Necci *et al.* 2017). MobiDB-lite was developed to improve the prediction of long intrinsic disorder regions by combining predictions from multiple tools to address these limitations (Necci *et al.* 2017, 2021a,b). The consensus was optimized on a PDB X-ray missing residue dataset. This approach minimized over- and under-prediction of disordered regions, achieving a balance that allowed MobiDB-lite to be effectively used for large-scale proteome annotation as available in the MobiDB database (Piovesan *et al.* 2025). The previous release, MobiDB-lite 3.0, added the classification of disorder subtypes based on sequence features (Das and Pappu 2013, Holehouse *et al.* 2017, Necci *et al.* 2021a,b). MobiDB-lite has been integrated into InterProScan (Jones *et al.* 2014) to maintain synchronization with major databases such as UniProtKB, InterPro, DisProt, and PDBe-KB (PDBe-KB Consortium 2022, Aspromonte *et al.* 2024, Blum *et al.* 2025, The UniProt Consortium 2025).

Recent research has shown that the ensemble properties of IDRs are linked to the biological function of the protein and may be predicted from the sequence (Lotthammer *et al.* 2024, Tesei *et al.* 2024). In the cellular context, the chain compaction and charge properties of IDRs may play central roles in function and affect interactions both within and between proteins.

MobiDB-lite 4.0 integrates the prediction of IDR compactness based on the apparent scaling exponent (*ν*) using a support vector regression (SVR) model developed by (Tesei *et al.* 2024). The software was used to generate predictions for the latest version of the MobiDB database (Piovesan *et al.* 2025). About 1.3 million proteins in MobiDB were found to have compact IDRs covering 15.9% of their residues, and 26 million proteins had extended IDRs covering 11.4% of their sequences. The MobiDB-lite 4.0 package was rewritten entirely to optimize execution time, making it ten times faster than the previous versions.

## 2 Implementation

As detailed in Necci *et al.* (2021a,b), MobiDB-lite is implemented in a two-step process. First, it computes a strict majority consensus among state-of-the-art predictors (i.e. more than 5 out of 8), namely ESpritz (DisProt, NMR, X-ray flavors), IUPred (short, long flavors), DisEMBL (HotLoop, Remarks465 flavors), and GlobPlot (Linding *et al.* 2003a,b, Dosztányi *et al.* 2005, Walsh *et al.* 2012). This consensus is then refined using a process similar to dilation-erosion morphological operations. The process iteratively refines disordered and ordered regions by converting short stretches (1–3 residues) based on their surrounding context. Structured stretches of up to 10 residues are reclassified as disordered if flanked by disordered regions of at least 20 residues on both sides. Finally, a length cutoff is applied to IDRs, and only those of at least 20 residues are kept. In the next stage, the predicted disordered regions are classified based on their structural and potential functional properties (i.e. polyampholyte, positive polyelectrolyte, and negative polyelectrolyte) (Das and Pappu 2013), enrichment in specific residues (i.e. cysteine-rich, proline-rich, glycine-rich, polar), or exhibiting low complexity (Wootton and Federhen 1993). This classification uses a sliding window of nine residues, with sub-regions reported if they are at least nine residues long.

MobiDB-lite 4.0 was enhanced to include annotations for “compact” and “extended” IDRs, corresponding to the ensemble compactness of disordered regions. This classification is based on the apparent scaling exponent (*ν*), predicted by a support vector regression (SVR) model developed by (Tesei *et al.* 2024). IDRs with *ν* ≤ 0.475 are labeled as “compact,” while those with *ν* > 0.55 are labeled as “extended.” These thresholds correspond to those separating the 5% most compact and 32% most expanded IDRs in the human proteome, respectively (Tesei *et al.* 2024).

In collaboration with InterPro, the MobiDB-lite package was rewritten for improved performance. Python2.7 dependency was removed, software design was simplified, and parallelization was shifted from individual disorder predictors to the protein level using multithreading instead of multiprocessing library. MobiDB-lite 4.0 was benchmarked against previous versions on five proteomes using single and multithreading (results at https://github.com/matthiasblum/idrpred). Processing the human proteome took 5 h and 45 min using a single thread and 42 min using eight threads with MobiDB-lite 4.0. Furthermore, the execution time for one million random UniParc (The UniProt Consortium 2025) sequences using 16 threads dropped from 80 h (v3.2.4) to 4 h and 42 min (v4.0). The time complexity of v4.0 using a single thread remains linear. The integration of the new “compact” and “extended” labels does not significantly impact execution time, as the classification is efficient and applied only to MobiDB-lite’s IDRs that contain at least 30 residues.

In the third edition of the Critical Assessment of Protein Intrinsic Disorder (CAID3) (Necci *et al.* 2021a,b, Del Conte *et al.* 2023), MobiDBlite 4.0 achieved an AUC of 0.797 in the Disorder-NOX category, placing it ahead of AlphaFold-pLDDT (AUC = 0.789) (Piovesan *et al.* 2022). The method displayed high precision in highly confident predictions. The full results of CAID3 are available at https://caid.idpcentral.org/challenge/results. Users can execute the software directly from the CAID prediction portal (Del Conte *et al.* 2023).

## 3 Use case

**Figure 1.**
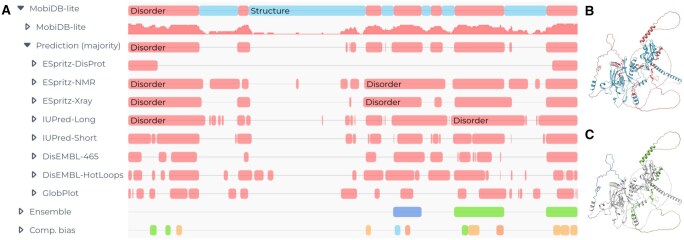
MobiDB-lite 4.0 predictions for protein C0NWB2 provided by the MobiDB database. (A) The MobiDB-lite’s results for protein C0NWB2. The disorder predictor takes the majority consensus among eight methods. The “Ensemble” track provides extended/compact annotations, while the “Comp. bias” track indicates sub-regions identified by MobiDB-lite. (B) The MobiDB-lite predictions on the AlphaFold2 structure of the same protein. (C) The extended and compact regions, colored green and blue, respectively.

MobiDB-lite 4.0 was used to predict disordered regions in the MobiDB database v6.1 (2024_07 release). Figure 1 illustrates the predictions for C0NWB2 (transcription factor Snf5p) in MobiDB. This protein is poorly characterized, and its only positional annotations in UniProtKB originate from MobiDB-lite. Among the 245 482 527 proteins in MobiDB, 57 172 035 (23.28%) contained at least one IDR covering 33.72% of their sequences. About 1.3 million proteins in MobiDB were found to have compact IDRs covering 15.9% of their residues, and 26 million proteins had extended IDRs covering 11.4% of their sequences. With the new ensemble feature, we find examples such as the C-terminal domain of CTR9 (Q6PD62, *ν*  =  0.412) and the *N*-terminal domain of FUS (P35637, *ν*  =  0.467), whereas among the expanded ones, we find BASP1 (P80723, *ν*  =  0.552) and prothymosin-α (P06454, *ν*  =  0.592).

In addition to identifying and characterizing the regions, MobiDB-lite 4.0 outputs were used to predict functional annotation for IDRs. This resulted in annotating 16 827 365 proteins with molecular function terms from Gene Ontology and 44 598 417 proteins with disorder function terms from Intrinsically Disordered Proteins Ontology (IDPO) (Aleksander *et al.* 2023, Aspromonte *et al.* 2024).

## 4 Conclusions

In this work, we described MobiDB-lite 4.0, which predicts intrinsic disordered regions and annotates them based on their compactness and sequence features. This functionality gives users profound insights into the biological roles of proteins. The software was restructured and the execution time is now ten times faster. MobiDB-lite is available as a docker container and integrated within InterProScan, making it a powerful tool for large-scale proteome-wide disorder annotation. It is periodically executed on all known protein sequences, and its predictions are integrated into MobiDB, InterPro, DisProt, PDBe-KB, and UniProtKB databases, among others.

Conflict of interest: None declared.

## Code availability

The MobiDB-lite 4.0 source code and a Docker container are available from the GitHub repository: https://github.com/BioComputingUP/MobiDB-lite.

## References

[btaf297-B1] Aleksander SA , Balhoff J, Carbon S et al; Gene Ontology Consortium. The gene ontology knowledgebase in 2023. Genetics 2023;224:iyad031. 10.1093/genetics/iyad031 PMC10158837 36866529

[btaf297-B2] Aspromonte MC , Nugnes MV, Quaglia F et al; DisProt Consortium. DisProt in 2024: improving function annotation of intrinsically disordered proteins. Nucleic Acids Res 2024;52:D434–41. 37904585 10.1093/nar/gkad928 PMC10767923

[btaf297-B4] Blum M , Andreeva A, Florentino LC et al InterPro: the protein sequence classification resource in 2025. Nucleic Acids Res 2025;53:D444–56. 39565202 10.1093/nar/gkae1082 PMC11701551

[btaf297-B5] Conte AD , Mehdiabadi M, Bouhraoua A et al Critical assessment of protein intrinsic disorder prediction (CAID)-results of round 2. Proteins Struct Funct Bioinf 2023;91:1925–34. 10.1002/prot.26582 37621223

[btaf297-B6] Das RK , Pappu RV. Conformations of intrinsically disordered proteins are influenced by linear sequence distributions of oppositely charged residues. Proc Natl Acad Sci USA 2013;110:13392–7. 23901099 10.1073/pnas.1304749110 PMC3746876

[btaf297-B7] Del Conte A , Bouhraoua A, Mehdiabadi M et al CAID prediction portal: a comprehensive service for predicting intrinsic disorder and binding regions in proteins. Nucleic Acids Res 2023;51:W62–69. 37246642 10.1093/nar/gkad430 PMC10320102

[btaf297-B8] Dosztányi Z , Csizmok V, Tompa P et al IUPred: web server for the prediction of intrinsically unstructured regions of proteins based on estimated energy content. Bioinformatics 2005;21:3433–4. 15955779 10.1093/bioinformatics/bti541

[btaf297-B9] Forman-Kay JD , Mittag T. From sequence and forces to structure, function, and evolution of intrinsically disordered proteins. Structure 2013;21:1492–9. 24010708 10.1016/j.str.2013.08.001 PMC4704097

[btaf297-B10] Holehouse AS , Das RK, Ahad JN et al CIDER: resources to analyze Sequence-Ensemble relationships of intrinsically disordered proteins. Biophys J 2017;112:16–21. 28076807 10.1016/j.bpj.2016.11.3200 PMC5232785

[btaf297-B11] Jones P , Binns D, Chang H-Y et al InterProScan 5: genome-scale protein function classification. Bioinformatics 2014;30:1236–40. 24451626 10.1093/bioinformatics/btu031 PMC3998142

[btaf297-B12] Linding R , Jensen LJ, Diella F et al Protein disorder prediction: implications for structural proteomics. Structure 2003a;11:1453–9. 14604535 10.1016/j.str.2003.10.002

[btaf297-B13] Linding R , Russell RB, Neduva V et al GlobPlot: exploring protein sequences for globularity and disorder. Nucleic Acids Res 2003b;31:3701–8. 12824398 10.1093/nar/gkg519 PMC169197

[btaf297-B14] Lotthammer JM , Ginell GM, Griffith D et al Direct prediction of intrinsically disordered protein conformational properties from sequence. Nat Methods 2024;21:465–76. 38297184 10.1038/s41592-023-02159-5 PMC10927563

[btaf297-B15] Necci M , Piovesan D, Clementel D et al MobiDB-lite 3.0: fast consensus annotation of intrinsic disorder flavors in proteins. Bioinformatics 2021a;36:5533–4. 33325498 10.1093/bioinformatics/btaa1045

[btaf297-B16] Necci M , Piovesan D, Dosztányi Z et al MobiDB-lite: fast and highly specific consensus prediction of intrinsic disorder in proteins. Bioinformatics 2017;33:1402–4. 28453683 10.1093/bioinformatics/btx015

[btaf297-B17] Necci M , Piovesan D, Tosatto SCE et al; DisProt Curators. Critical assessment of protein intrinsic disorder prediction. Nat. Methods 2021b;18:472–81. 33875885 10.1038/s41592-021-01117-3 PMC8105172

[btaf297-B18] Pappu RV , Cohen SR, Dar F et al Phase transitions of associative biomacromolecules. Chem Rev 2023;123:8945–87. 36881934 10.1021/acs.chemrev.2c00814 PMC11513790

[btaf297-B19] PDBe-KB Consortium. PDBe-KB: collaboratively defining the biological context of structural data. Nucleic Acids Res 2022;50:D534–42. 34755867 10.1093/nar/gkab988 PMC8728252

[btaf297-B20] Piovesan D , Monzon AM, Tosatto SCE et al Intrinsic protein disorder and conditional folding in AlphaFoldDB. Protein Sci 2022;31:e4466. 36210722 10.1002/pro.4466 PMC9601767

[btaf297-B21] Piovesan D , Del Conte A, Mehdiabadi M et al MOBIDB in 2025: integrating ensemble properties and function annotations for intrinsically disordered proteins. Nucleic Acids Res 2025;53:D495–503. 39470701 10.1093/nar/gkae969 PMC11701742

[btaf297-B22] Silva A , Almeida B, Fraga JS et al Distribution of amyloid-like and oligomeric species from protein aggregation kinetics. Angew Chem Int Ed Engl 2017;56:14042–5. 28906069 10.1002/anie.201707345

[btaf297-B23] Tesei G , Trolle AI, Jonsson N et al Conformational ensembles of the human intrinsically disordered proteome. Nature 2024;626:897–904. 38297118 10.1038/s41586-023-07004-5

[btaf297-B27] The UniProt Consortium. UniProt: the Universal Protein Knowledgebase in 2025. Nucleic Acids Res 2025;53:D609–17. 39552041 10.1093/nar/gkae1010 PMC11701636

[btaf297-B24] Walsh I , Martin AJM, Di Domenico T et al ESpritz: accurate and fast prediction of protein disorder. Bioinformatics 2012;28:503–9. 22190692 10.1093/bioinformatics/btr682

[btaf297-B25] Wootton JC , Federhen S. Statistics of local complexity in amino acid sequences and sequence databases. Comput. Chem 1993;17:149–63.

[btaf297-B26] Wright PE , Dyson HJ. Intrinsically disordered proteins in cellular signalling and regulation. Nat Rev Mol Cell Biol 2015;16:18–29. 25531225 10.1038/nrm3920 PMC4405151

